# Formulation and In Vivo Evaluation of a Solid Self-Emulsifying Drug Delivery System Using Oily Liquid Tocotrienols as Model Active Substance

**DOI:** 10.3390/pharmaceutics13111777

**Published:** 2021-10-25

**Authors:** You Zhuan Lee, Eng Kwong Seow, Sheau Chin Lim, Kah Hay Yuen, Nurzalina Abdul Karim Khan

**Affiliations:** School of Pharmaceutical Sciences, Universiti Sains Malaysia, George Town 11800, Penang, Malaysia; youzhuanlee@gmail.com (Y.Z.L.); ekseow93@gmail.com (E.K.S.); siao_c@yahoo.com (S.C.L.); khyuen@usm.my (K.H.Y.)

**Keywords:** self-emulsifying drug delivery system, poorly water-soluble drugs, solid dosage forms, in vivo oral bioavailability, pharmacokinetic, tocotrienols

## Abstract

Self-emulsifying drug delivery systems (SEDDS) can improve the oral bioavailability of poorly water-soluble drugs. Solid self-emulsifying drug delivery systems (s-SEDDS) offer several advantages including improved drug stability, ease of administration, and production. Most compounds employed in developing s-SEDDS are solid in nature, with a high amount of surfactants added. The aim of this study was to develop an s-SEDDS using a tocotrienol-rich fraction (TRF) as the model liquid active substance via a simple adsorption method. The solid formulation was developed using magnesium aluminosilicate as the carrier with 70% TRF and 30% surfactants (poloxamer and Labrasol^®^). The formulation showed good self-emulsification efficiency with stable emulsion formed, excellent powder flowability, and small emulsion droplet size of 210–277 nm. The s-SEDDS with combined surfactants (poloxamer and Labrasol^®^) showed a faster absorption rate compared to preparations with only a single surfactant and enhanced oral bioavailability (3.4–3.8 times higher) compared to the non-self-emulsifying oily preparation when administered at a fasted state in rats. In conclusion, an s-SEDDS containing a high amount of TRF was successfully developed. It may serve as a useful alternative to a liquid product with enhanced oral bioavailability and the added advantage of being a solid dosage form.

## 1. Introduction

It is estimated that 70 to 90% of newly discovered drugs suffer from poor aqueous solubility [1,2]. Poor water solubility is one of the critical limiting factors in affecting dissolution and consequently drug absorption and oral bioavailability [3]. A self-emulsifying drug delivery system (SEDDS) provides an effective method to improve the bioavailability of poorly water-soluble compounds. An SEDDS is an isotropic mixture of oil and surfactant(s) that is able to produce fine dispersion upon aqueous dilution in the gastrointestinal tract, which is aided by the gentle agitation provided by the gut motility [4,5]. It has the attributes of emulsions in terms of enhancing oral drug bioavailability, with an improved physical stability as the aqueous phase is only introduced in the gastrointestinal tract upon ingestion [5].

Due to the nature of excipients used, conventional SEDDS are usually in liquid form and typically encapsulated in soft or hard gelatin capsules [6]. Some SEDDS liquid products suffer from chemical and physical stability issues such as content migration or drug precipitation [7,8,9]. In addition, liquid formulations may have material compatibility issues, potential leakage problem, and migration of excipients into a capsule shell [10]. Moreover, liquid filling into capsules requires specialised technology and processing equipment [6,10].

The solidification of formulations is one feasible approach to improve the stability and compatibility issues associated with the liquid systems [9]. Solid formulations are also easier to be transported and stored compared to liquid formulations that are bulky and susceptible to stability and microbial challenges. Solid formulations may also enable simpler handling and manufacturing processes [8,9]. Thus, solid self-emulsifying drug delivery systems (s-SEDDS) possess the advantages of SEDDS together with that of solid dosage forms [8].

However, s-SEDDS may have several limitations. Complex processes (spray drying, freeze drying, rotary evaporation) are usually involved, and these approaches often produce products with low yield [11], low final drug loading [12,13,14,15,16,17], poor powder flow [18], and require the usage of volatile solvents such as chloroform and methanol [19]. The solidification of SEDDS can also be achieved using thermoplastic surfactants or lipids via melt granulation or pelletisation. High shear mixing of molten binders with drug and excipients was applied to obtain granules or pellets [10,20]. The main limitation of this method is the relatively high temperature used in melting, which may cause chemical degradation or affect the crystallinity of the ingredients [21,22]. If high amounts of liquid are loaded onto meltable binders, only semi-solids could be achieved [8,23]. S-SEDDS can also be obtained by adsorption onto solid carriers [8,10]. This simple process involves addition of the liquid formulation onto solid carriers. The free-flowing powder obtained may be filled into capsules or compressed into tablets [8]. However, high liquid loading may lead to powder or granules that are too soft or have poor flowability. More excipients added to improve the powder flow properties may dilute the formulations [24]. In addition, an incomplete release of adsorbed compounds has been reported when some solid carriers were used [20,25].

Many studies on s-SEDDS utilised high concentrations of surfactants and co-surfactants of more than 80% [12,13,14,16,17,26] and 50 to 79% [27,28,29,30,31]. A high quantity of surfactants may cause gastrointestinal irritation [5,8]. In addition, most research on s-SEDDS focused on improving the delivery of solid compounds such as docetaxel, celecoxib, tacrolimus, and itraconazole [16,27,32,33], to name a few. There might be a research gap in converting liquid active compounds into s-SEDDS.

The aim of this study was to develop an s-SEDDS with high liquid drug loading and minimum surfactants through a simple adsorption method to achieve efficient self-emulsification and free-flowing powder. Thus, an attempt was made in the present study to formulate an s-SEDDS for a liquid active compound using a tocotrienol mixture as the model active substance. Tocotrienols as part of the vitamin E family include alpha-, beta-, delta-, and gamma-tocotrienol. Tocotrienols reportedly possess biological activities including antioxidant [34,35], anticancer [36,37], neuroprotective [38,39], and cardioprotective properties [40], among others. Similar to other fat-soluble vitamins, the bioavailability of tocotrienols is poor and variable [41,42], making tocotrienols the suitable model active substance for s-SEDDS. The first part of the investigation was to evaluate and choose a suitable solid carrier for the s-SEDDS that would be able to hold high amounts of liquid. Then, s-SEDDS containing various combinations of oil and surfactants were tested for their self-emulsifying efficiency before analysing the powder flow properties, in vitro drug release, and emulsion droplet size. Subsequently, two in vivo studies were carried out to determine the oral bioavailability of the s-SEDDS with varying surfactant compositions and also to compare the absorption of the s-SEDDS with those of the commercial liquid SEDDS, Tocovid Suprabio™, (Ipoh, Malaysia), and a non-self-emulsifying oily preparation.

## 2. Materials and Methods

### 2.1. Materials

A tocotrienol-rich fraction (TRF) was obtained from Excelvite (Ipoh, Malaysia). According to the manufacturer’s certificate of analysis (COA), TRF contained 7.1%, 20.6%, and 13.9% of delta-, gamma-, and alpha-tocotrienol, respectively. The TRF content was periodically analysed to ensure its level according to the COA prior to use. The rest of the TRF consisted mainly of alpha-tocopherol, palm olein, plant squalene, and sterol complex, with traces of carotenoid complexes. The excipients used in the experiments were generously provided by Hovid Ltd., Ipoh, Malaysia. They were Aerosil^®^ 200 (colloidal silicon dioxide; Evonik, Essen, Germany), Avicel^®^ (Microcrystalline cellulose (MCC) PH 101, PH 102, PH 112, and PH200; FMC Biopolymer, Philadelphia, PA, USA), StarCap^®^ (corn starch; Colorcon, Dartfort, UK), Fujicalin^®^ (dibasic calcium phosphate; Fuji Chemical Industry Co., Toyama, Japan), Klucel™ (hydroxypropyl cellulose HPC LF and EXF; Ashland, Wilmington, DE, USA), Methocel™ (hydroxypropyl methylcellulose HPMC E5, E15, K100 PLV, K4M; Colorcon, Dartfort, UK), GranuLac^®^ 100 (lactose monohydrate; MEGGLE, Wasserburg, Germany), LYCATAB^®^ (maltodextrin; Roquette, Lestrem, France), Starch 1500^®^ (pregelatinised starch; Colorcon, Dartfort, UK), Aqualon™ (sodium carboxymethylcellulose (CMC), Ashland, Wilmington, DE, USA), Ac-Di-Sol^®^ (croscarmellose sodium; FMC Biopolymer, Philadelphia, PA, USA), Labrasol^®^ (caprylocaproyl macrogol-8 glycerides; Gattefossé, Saint-Priest, France), and sodium lauryl sulfate (SLS; BASF, Los Angeles, CA, USA). Poloxamer 188 was obtained from BASF (Los Angeles, CA, USA). Neusilin US2 (magnesium aluminosilicate) was obtained from Fuji Chemical Industry Co. (Toyama, Japan). Solvents, either analytical or HPLC grades, were from Merck Life Sciences (Darmstadt, Germany). Tocovid Suprabio™ was obtained from Hovid Ltd., Ipoh, Malaysia.

### 2.2. Preparation and Assessment of s-SEDDS

#### 2.2.1. Solid Carrier Selection

The liquid TRF was added gradually to 1 g of different solid carriers and mixed using a mortar and pestle until a homogeneous powder was obtained. The addition of liquid was discontinued once a lump or paste was formed. Then, the amount of the liquid adsorbed by the solid carriers was recorded by calculating the weight difference in the liquid TRF used in the addition.

#### 2.2.2. Preparation of s-SEDDS Tocotrienol Powder

Liquid self-emulsifying mixtures were first prepared using TRF and two surfactants: Labrasol^®^ and poloxamer 188, which are in liquid and solid state at room temperature, respectively. Each ingredient was carefully weighed out in a test tube and melted at 60 ± 2 °C in a water bath for about 15 min and vortex-mixed every 5 min for 30 s to ensure all ingredients were mixed homogenously.

Then, the s-SEDDS powders were prepared by the adsorption method via a combination of wet and melt granulation. The molten liquid mixtures (containing TRF, Labrasol^®^, and/or poloxamer 188) were gradually added to the solid excipients (68% liquid self-emulsifying mixture, 28% Neusilin, 2% Ac-Di-Sol, and 2% SLS) and mixed well until homogenous using a mortar and pestle.

#### 2.2.3. Assessment of Self-Emulsification Properties

The test apparatus used for evaluating the self-emulsifying efficiency was based on the one adopted by Julianto [43]. The apparatus consisted of a light source, a paddle stirrer, a 250 mL beaker, a current relay, and a phototransistor, which were placed in-line accordingly. The light source was from a 40-watt bulb, giving light intensity of about 1000 lux that passes through the glass beaker filled with 250 mL of distilled water. The phototransistor was connected to a current relay and a stopwatch. The paddle stirrer was set to rotate at 100 rpm.

To assess the self-emulsifying properties of the formulations, a syringe containing 0.5 mL of the liquid formulation or 700 mg of the solid preparation was placed 1 cm below the water surface in the beaker prior to injection. When the sample was introduced into the 250 mL of distilled water (37 °C), the stopwatch was initiated simultaneously. The paddle stirrer provided gentle agitation to the contents in the beaker. If an emulsion was formed and was able to block the light that passed through the beaker initially, the phototransistor would not be able to detect any light, and the stopwatch would be triggered to stop via the current relay. The time recorded was used to compare the self-emulsifying efficiency among the different samples. All samples were allowed a maximum time of 5 min if the stopwatch was not triggered to stop.

The self-emulsifying properties of the formulations were also assessed visually based on the quality of the emulsions formed. They were categorised into different groups based on the visual grading system adapted from Khoo et al. [44]: Grade A to indicate a rapidly forming emulsion (within 2 min) with high cloudiness to block the scattering light and trigger the stopwatch; Grade B to indicate a rapidly forming emulsion (within 2 min) with less cloudiness and unable to block the light as oil contents were deemed to be partially solubilised by the surfactants; Grade C to indicate a slow forming coarse emulsion (beyond 2 min) that was slightly oily; Grade D to indicate a minimal emulsion formed with large oil droplets on the surface of the distilled water; and Grade E to indicate a formulation that does not emulsify, with oil layering on top of the water surface.

### 2.3. Characterisation of the Optimised s-SEDDS

#### 2.3.1. Evaluation of Powder Flow Properties

Powder flowability was determined using Carr’s compressibility index (CI), the Hausner ratio (HR), and the angle of repose. CI and HR were derived from bulk and tapped density. The angle of repose was determined using the funnel method [45,46]. Bulk and tapped density were measured using the Digital Bulk Density Apparatus (Edutek Instrumentation, India) with a 25-mL glass measuring cylinder. The tap height was adjusted to 3 mm. Powder (5 g) was weighed accurately and poured into a 25 mL graduated measuring cylinder, and the bulk volume was recorded. The same cylinder was placed on the tapped density measuring apparatus and tapped 200 times, with 100 times increment until no further change in volume was observed. The tapped volume was recorded directly from the cylinder marks.

#### 2.3.2. Release Studies of Mixed Tocotrienols from s-SEDDS

The s-SEDDS (100 mg) were carefully weighed out into a 50 mL centrifuge tube and added with 50 mL of buffer solution (pH 1.2, 4.5 or 6.8). The contents in the centrifuge tube were rotated for 3 h to ensure complete release, before it was centrifuged for 5 min at 450 RCF (relative centrifugal force) to precipitate the solids [25]. Supernatant was carefully collected and diluted before injecting into the HPLC. The extent of content release was calculated in terms of percentage detected in the media compared to the amount added.

#### 2.3.3. HPLC Analysis

The assays of tocotrienols (delta, gamma, and alpha isomers) were determined using high-performance liquid chromatography (HPLC) method as reported and validated by Yap et al. [47]. The HPLC system consisted of a Waters 2695 Alliance^®^ Separation Module (Waters, Milford, MA, USA) and Waters 2475 Fluorescent Detector (Waters, Milford, MA, USA). The detector operated at an excitation wavelength of 296 nm and emission wavelength of 330 nm, with sensitivity set at 10,000 EUFS. A Hichrom C18 (4 µm, 250 × 4.6 mm i.d.) analytical column (Reading, Berkshire, UK) was fitted to a refillable guard column (2 mm × 2 cm) (Upchurch Scientific, Oak Harbor, WA, USA) packed with Whatman Partisil-10 ODS-3 (Clifton, NJ, USA). The mobile phase used was pure methanol, and the system was set to operate at 25 °C. The injection volume was set at 50 µL. Analyses were carried out at a flow rate of 1.0 mL/min, and the samples were quantified using peak area. The chromatograms are available in the Supplementary Materials (Figures S1 and S2).

#### 2.3.4. Stability Evaluation of Emulsion Product

The physical stability of the emulsion products formed after standing for 2 h at room temperature (25 °C) was evaluated. An amount (700 mg) of each formulation was dispersed into 250 mL of distilled water and stirred using a paddle stirrer rotating at 100 rpm for 10 min to obtain an emulsion product. Then, approximately 10 mL of the emulsion formed was immediately transferred into a test tube and allowed to stand. After two hours, samples were visually inspected. The physical stability of the emulsion products based on visual observation was categorised as no separation, slight creaming, creaming, and complete separation of phases. All experiments were conducted in triplicate.

#### 2.3.5. Droplet Size Analysis of the Emulsion Products

The formulations (100 mg) were weighed carefully into 50 mL centrifuge tubes and added with 50 mL of filtered (0.22-µm) distilled water. The tubes were rotated for 1 h and then centrifuged at 450 RCF for 5 min to remove the water-insoluble Neusilin [25]. The supernatant collected from each formulation was diluted (0.5 mL in 45 mL of filtered distilled water) prior to analysis. Emulsion droplet size was measured by means of photon correlation spectroscopy (PCS) using the Malvern Zetasizer 1000HS (Worcestershire, UK). For all procedures involved in the measurements of emulsion droplet size, filtered distilled water was used to ensure a count rate value of below 3 K at a 90° scattering angle. All samples were diluted to obtain a count rate of 200–300 K. Ten measurements were taken as set by the method of the software.

### 2.4. In Vivo Oral Bioavailability Studies

Two separate experiments were conducted to study the relative oral bioavailability of the tocotrienol preparations. Study I compared the absorption between the s-SEDDS containing 70% TRF and different composition of surfactant(s) of either Labrasol^®^ and/or poloxamer; while Study II compared that between the s-SEDDS, commercial liquid SEDDS, Tocovid Suprabio™, and non-self-emulsifying preparation.

#### 2.4.1. Animals

The study protocol was approved by the Institutional Animal Care and Use Committee of Universiti Sains Malaysia (USM IACUC) (No. of Animal Ethics Approval: USM/IACUC/2017/(106)(865). Male Sprague–Dawley rats (weighed 259.5 ± 8.3 g for Study I and 272.6 ± 23.9 g for Study II) were obtained from the Animal Research and Service Centre of Universiti Sains Malaysia. The rats were housed under normal laboratory conditions with free access to standard rodent diet and water.

#### 2.4.2. Preparation of Various Mixed Tocotrienol Preparations

The preparation of s-SEDDS powder B1, B2, and B3 has been described in the earlier section (2.2.2). The solid preparation was made from liquid mixture containing 70% TRF and 30% surfactant(s). The solid preparation made from liquid mixture B2 had combined surfactants (15% Labrasol^®^ and 15% poloxamer), while that from B1 and B3 consisted of only one of either surfactant (30% Labrasol^®^ or 30% poloxamer). Each 100 mg of s-SEDDS had 3.9, 8.9, and 5.8 mg of delta-, gamma-, and alpha-tocotrienol, respectively.

The commercial liquid SEDDS Tocovid Suprabio™ soft gelatin capsules were cut open, and their contents were collected in an amber jar. The liquid was stirred for 20 min to obtain a homogenous mixture using a magnetic hot plate stirrer. According to the product label, one 600 mg capsule of the Tocovid Suprabio™ contains 50 mg of mixed tocotrienols (consisting of 6.42 mg, 28.20 mg, and 15.38 mg of delta-, gamma-, and alpha-tocotrienol, respectively).

TRF, which contained 8.1%, 18.6%, and 12.1% of delta-, gamma-, and alpha-tocotrienol respectively, was considered concentrated, and it was difficult to weigh out the tocotrienol dose needed. Thus, TRF was diluted with soya oil (the final mixture consisted of 25% TRF and 75% soya oil) to give final delta, gamma-, and alpha-tocotrienol concentration of 2.0 mg, 4.7 mg, and 3.0 mg, respectively, per 100 mg final oily suspension. The mixture was stirred for 20 min to obtain homogeneity using a magnetic hot plate stirrer.

#### 2.4.3. Experimental Protocol

The experiments were conducted using adult male Sprague–Dawley rats according to a three-period, three-sequence crossover study with a one-week washout period between the phases. The rats were randomly assigned into 3 groups of 2 rats each and administered the preparations according to sequence shown in Table 1. The rats were fasted for at least 8 h before drug administration and during the 12 h study period. Free access to water was allowed throughout the study period except for 2 h after dose administration. The dose of these preparations was fixed at 20 mg/kg of mixed tocotrienols per rat. Each formulation was administered by oral gavage. Each formulation (solid or liquid) was carefully weighed into the dosing syringe used for oral administration. Immediately before dosing, 0.5 mL of distilled water was drawn into the syringe. Then, the content was mixed by shaking prior to orally administering to the rats. Approximately 0.3 mL of blood samples were collected from the rat’s tail vein into heparinised microcentrifuge tubes according to the sampling interval at 0 (before dosing), 0.5, 1, 1.5, 2, 3, 4, 6, 8, and 12 h post oral dosing. After centrifugation of the blood samples for 20 min at 12,800 RCF, the supernatant (plasma) was carefully collected and stored at −20 °C until analysis.

#### 2.4.4. Analysis of Plasma Delta-, Gamma- and Alpha-Tocotrienol

A 100 µL aliquot of rat plasma sample was measured into a microcentrifuge tube and deproteinised by adding 200 µL of a mixture of acetonitrile: tetrahydrofuran (3:2, *v*/*v*). Then, the mixture was vortex-mixed for 2.5 min using a vortex mixer and centrifuged at 12,800 RCF for 20 min. A 50 µL aliquot of the supernatant was injected into the HPLC system according to method described in Section 2.3.3 [47].

#### 2.4.5. Data and Pharmacokinetic Analysis

The oral bioavailability of the three tocotrienol formulations was compared using pharmacokinetic parameters calculated from the plasma concentration-time data. The parameters were area under the plasma concentration-time curve from time zero to the last sampling point (AUC_0-12h_), maximum plasma concentration (C_max_), and time to reach maximum plasma concentration (T_max_). The values of C_max_ and T_max_ were obtained directly from the plasma values. The AUC_0-12h_ was calculated using the trapezoidal formula [48]. For Study 2, since the three products have differences in the content of the three tocotrienol isomers, the plasma concentration values were normalised to that of the TRF preparation.

#### 2.4.6. Statistical Analysis

The AUC_0-12h_ and C_max_ values obtained from the study comparing the different tocotrienol formulations were analysed using an analysis of variance (ANOVA) procedure appropriate for a crossover study design [49]. The AUC_0-12h_ and C_max_ values were logarithmic transformed prior to analysis. When a statistically significant difference was detected from the ANOVA procedure, Tukey’s test for pairwise comparison was carried out. T_max_ values from the three preparations were analysed using Friedman’s test. A statistical significance was indicated when *p <* 0.05. Microsoft Excel (Microsoft Corp., Redmond, WA, USA) and SPSS (v 23.0, IBM Corp., Armonk, NY, USA) were used for statistical analysis.

## 3. Results

### 3.1. Solid Carrier Selection

**Figure 1:**
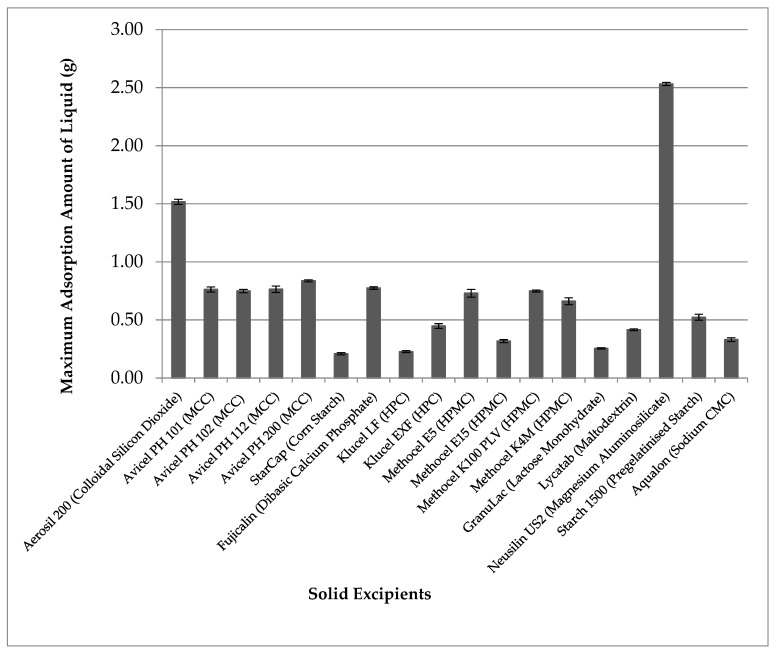
The maximum adsorption amount of liquid tocotrienol-rich fraction (TRF) on 1 g of different solid excipients (mean ± SD, n = 3).

Figure 1 shows the results of the maximum adsorption amount of liquid TRF onto 1 g of different solid pharmaceutical excipients. Among various carriers tested, the silica type of excipients Aerosil^®^ (silicon dioxide) and Neusilin US2 (magnesium aluminosilicate) showed higher loading capacity at more than 1 g of liquid per 1 g of solids. Among the two, Neusilin was able to carry more, at 2.5 times liquid to the amount of solid carrier. Thus, Neusilin was selected as the solid carrier to use in subsequent formulations.

### 3.2. Assessment of Self-Emulsification Properties

The results of visual assessment and self-emulsification efficiency studies of various liquid and solid SEDDS prepared using TRF, poloxamer 188, and Labrasol^®^ are shown in Table 2. Satisfactory emulsions with an emulsification time of less than 15 s were obtained when lower amounts of TRF and high amounts of surfactant were used. As the TRF component in the mixture was further increased to 80%, most preparations could only generate coarse or poorly formed emulsions. Most results were comparable to that the commercial liquid SEDDS product Tocovid Suprabio™, which was able to produce grade A emulsion in 5.1 ± 0.3 s. As the target was to produce solid formulations with high liquid load, more in-depth characterisation was conducted for solid formulations with 70% TRF.

### 3.3. Characterisation of the Optimised s-SEDDS

#### 3.3.1. Evaluation of Powder Flow Properties

Neusilin was tested for its micromeritic properties with bulk and tapped density to be 0.1670 ± 0.0002 and 0.1755 ± 0.0021 g/mL, respectively. Carr’s compressibility index and the Hausner ratio were calculated to be 4.86 ± 1.20 and 1.05 ± 0.01. Meanwhile, the angle of repose tested was 22.39° ± 1.02°, which was in the same ‘excellent’ category, as described in the US Pharmacopoeia [45]. Table 3 shows the flow properties of the s-SEDDS. From visual inspection, all powders were dry and free flowing. All batches of solid powder prepared were in the good to excellent category as described in the US Pharmacopoeia [45]. The figure of s-SEDDS B2 is presented in the Supplementary Materials (Figure S3).

#### 3.3.2. Release Studies of Mixed Tocotrienols from s-SEDDS

**Figure 2:**
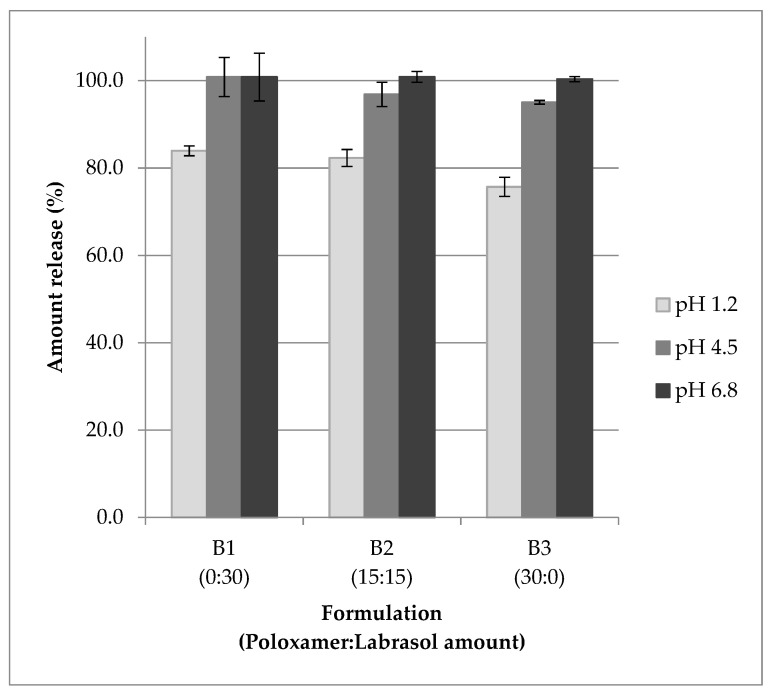
Amount of mixed tocotrienols release from s-SEDDS containing 70% TRF and 30% mixed surfactants in different pH (mean ± SD, n = 3).

Figure 2 shows the amount of tocotrienols released from formulation containing 70% TRF and 30% surfactants. The content release levels of mixed tocotrienols were 76 to 100% at different pH, with lower levels observed in pH 1.2. From the results obtained, it could be deduced that the tocotrienols could be readily released for absorption when the products are administered orally.

#### 3.3.3. Stability Evaluation of Emulsion Product

Table 4 shows the results for physical stability of the emulsion product formed from the s-SEDDS preparations B1, B2, and B3. From visual observation, the solid preparations produced emulsions that were evenly cloudy with slight sedimentation of the solid powder at the bottom of the test tubes after two hours standing. The solid powder observed at the bottom of the sample tubes was Neusilin, as it is insoluble in water.

#### 3.3.4. Droplet Size Analysis of the Emulsion Products

The emulsion droplet size and their polydispersity index (PDI) values for formulations containing 70% TRF and 30% surfactant(s) were in the range of 210 to 277 nm (Table 4). The PDI values were below 0.60 and demonstrated a decreasing trend as the amount of Labrasol^®^ was reduced.

### 3.4. In Vivo Oral Bioavailability Studies

#### 3.4.1. In Vivo Evaluation of Different s-SEDDS Mixed Tocotrienol Formulations

**Figure 3:**
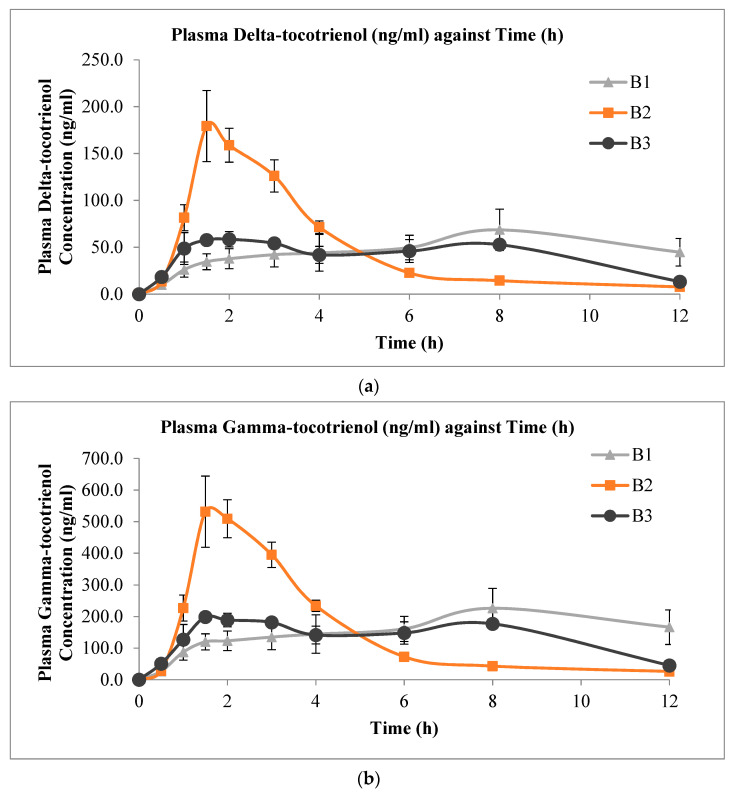

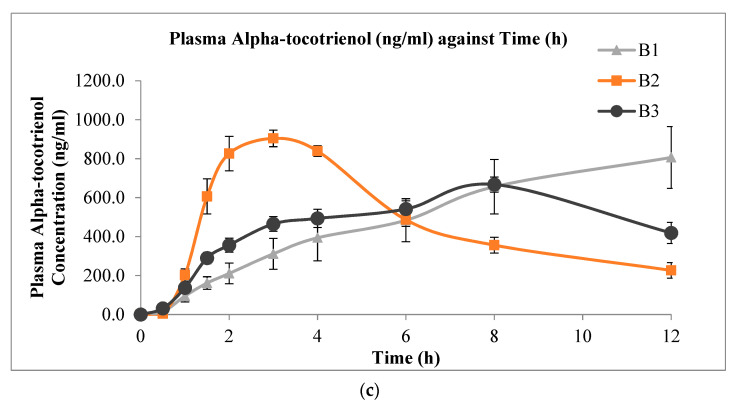
Mean plasma concentration versus time profiles (mean ± SEM, n = 6) of (**a**) delta-tocotrienol; (**b**) gamma-tocotrienol; and (**c**) alpha-tocotrienol after oral administration of 20 mg/kg mixed tocotrienols in s-SEDDS containing 70% TRF and 30% surfactant(s).

The mean plasma concentration versus time profiles of delta-, gamma-, and alpha-tocotrienol administered from the three s-SEDDS with varying surfactant compositions are shown in Figure 3. It is apparent from the profiles that the formulation with combined surfactants (formulation B2) had a faster rate of absorption, as indicated by the more rapid increase in plasma tocotrienol levels. Formulations with combined surfactants showed higher plasma tocotrienol concentrations compared to that of the other two solid formulations that contained a single surfactant up to the 6 h sampling point. At 6 to 12 h, the plasma levels of the formulation with combined surfactants remained slightly lower than the other two formulations. Table 5 shows the corresponding values of C_max_, T_max_, and AUC_0-12h_ from the experiments. The individual numerical values are presented in the Supplementary Materials (Tables S1–S3). Despite the significant differences in C_max_ and T_max_ among formulations, there appeared to be no statistically significant difference in the AUC_0-12h_ values among the three solid formulations for all three tocotrienol isomers.

#### 3.4.2. In Vivo Evaluation of s-SEDDS Versus Liquid SEDDS and Non-Self-Emulsifying Preparations of Tocotrienols

**Figure 4:**
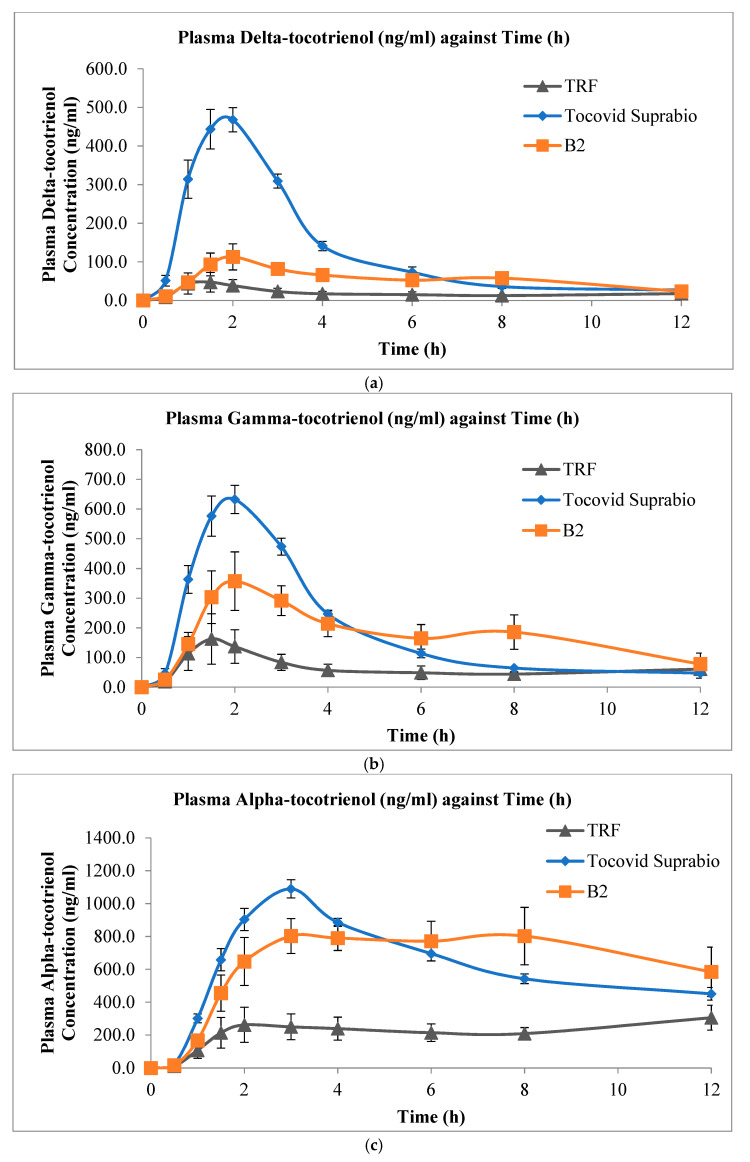
Mean plasma concentration versus time profiles (mean ± SEM, n = 5) of (**a**) delta-tocotrienol; (**b**) gamma-tocotrienol; and (**c**) alpha-tocotrienol after oral administration of 20 mg/kg mixed tocotrienols in three different tocotrienol preparations: the non-self-emulsifying oily preparation (TRF), Tocovid Suprabio™, and B2.

Figure 4 shows the mean plasma delta-, gamma-, and alpha-tocotrienol concentration versus time profiles of the three tocotrienol preparations, while Table 6 shows the corresponding values of C_max_, T_max_, and AUC_0-12h_. The individual numerical values are presented in the Supplementary Materials (Tables S4–S6). The oral bioavailability of delta-, gamma-, and alpha-tocotrienol from the self-emulsifying formulations, both solid and liquid, was higher than that of the non-self-emulsifying preparation. In addition, the liquid SEDDS Tocovid Suprabio™ appeared to have a faster rate of absorption, as seen from the sharper rise of the plasma tocotrienol level from their baseline level. This was followed by the s-SEDDS B2 and then the non-self-emulsifying formula.

A statistically significant difference was observed for the AUC_0-12h_ values (*p <* 0.05) between the SEDDS (both B2 and Tocovid Suprabio™) and the oily non-self-emulsifying liquid for all three tocotrienol isomers. The AUC_0-12h_ for all three tocotrienol isomers administered in both the solid B2 and liquid SEDDS Tocovid Suprabio™ formulations were 3.4 to 3.8 and 3.3 to 8.6 times higher than that of the oily non-self-emulsifying preparation, respectively. Between the solid B2 and liquid SEDDS Tocovid Suprabio™, no statistically significant difference was noted in both the AUC_0-12h_ and C_max_ values, except for delta-tocotrienol. Based on the AUC_0-12h_ values, the oral bioavailability from B2 was slightly lower than that of Tocovid Suprabio™ (with median of about 0.75) but higher than that of the non-self-emulsifying lipid preparation (median of about 3.6).

## 4. Discussion

### 4.1. Solid Carrier Selection

The ideal solid carrier should have the desirable characteristics including the ability to hold a large quantity of liquid to permit high loading of liquid active compounds. TRF was used as the liquid to select the solid carriers with the highest liquid load instead of the SEDDS during formulation optimisation, as the latter consisted of solid surfactants in different ratios that would affect the liquid load. Results from the present study show that silicate type of excipients (silicon dioxide and magnesium aluminosilicate) were able to hold the highest amount of liquid. Silicon dioxide exists as loose aggregates of very fine, nanosize particles, and the liquid adsorbed would spread in between particles [50]. On the other hand, Neusilin US2 (magnesium aluminosilicate) has a larger particle size and highly porous surfaces, with relatively large pores of up to 1 µm in diameter [51]. The adsorbed liquid not only spread on the surface of particles but also into the channels of the macropores of the silicate. This may explain Neusilin’s ability to hold high amounts of liquid [51]. Thus, Neusilin was selected as the ideal solid carrier for subsequent studies.

### 4.2. Assessment of Self-Emulsification Properties

Due to their amphiphilic nature, surfactants have the ability to solubilise high amounts of hydrophobic substances [52]. Surfactants act by reducing the interfacial tension between oil and water, thus stabilising the droplet phase during the emulsification process [53]. Two surfactants were used to prepare the liquid SEDDS before adsorption to a solid carrier, as it has been demonstrated that a mixture of the two surfactants may enhance the solubilisation efficiency of the compound, compared to when a single surfactant was used, in accordance to what was observed by other researchers [53,54,55]. In addition, a mixture of surfactants may reduce the total amount of surfactants needed to produce an efficient SEDDS [54].

It has also been studied that high hydrophile–lipophile balance (HLB) blends of surfactants have better solubilising properties compared to mixtures that have lower HLB values [56]. The present study combined Labrasol^®^ (HLB value of 12) and poloxamer 188 (HLB value of 29), which has a calculated HLB value of 20.5 [57], and these may further contribute to the solubilisation of the TRF. Poloxamer 188 has an added advantage of being solid at room temperature (melting point 52 to 57 °C), hence reducing the burden of solid carriers and permitting higher loading of the liquid active compound.

Observations showed that the time taken for emulsion to be successfully generated was increased with increasing amounts of poloxamer. This could be due to the higher viscosity induced by poloxamer in the liquid mixtures, which required greater shear force for dispersion in the aqueous medium, thus resulting in a longer emulsification time. However, this observation was not seen in the solid formulations. When the liquid formulations comprising poloxamer and Labrasol^®^ were added to the solid carrier and mixed homogenously, final products in powder form were obtained. The liquid mixtures, including polaxamer, were forced to disperse evenly and adsorbed onto the solid. This has greatly enhanced the surface area of the mixture, leading to an improvement in emulsification time compared to when the formulations were in molten liquid form [58]. The addition of SLS and Ac-Di-Sol^®^ (croscarmellose sodium) contributed to further improvements of emulsification efficiency. Highly hydrophobic compounds such as the TRF and silica carriers were not wetted easily by physiological fluids. Croscarmellose sodium has good hydrophilicity and facilitates fast water-wicking action [59]. SLS has frequently been used as a wetting agent to accelerate drug release and dissolution [59,60].

### 4.3. Characterisation of the Optimised s-SEDDS

Powder flow is a key requirement for efficient pharmaceutical manufacturing, as it determines the even mixing, packaging, tabletting, and capsule-filling process [46]. One of the limitations of loading a high amount of liquid SEDDS onto solid adsorbents is the potential poor flow of the final powder preparation [61]. The average particle size of Neusilin US2 from the literature and technical documents was 80–106 µm, with the appearance of white granules [62,63]. In the present study, the s-SEDDS product appeared slightly coarser but still in the granule form. The density of the s-SEDDS was more than doubled compared to Neusilin after the addition of liquid mixture with minimal changes to the appearance and still retaining the ‘excellent’ powder flow properties as per Neusilin based on the angle of repose, Carr’s compressibility index, and Hausner ratio. This is likely due to the Neusilin US2′s larger particle size (in granules rather than powder) and highly porous nature whereby liquids added were adsorbed and localised in the pores, minimising the particle agglomeration caused by interparticulate liquids [64]. Considering the high amounts of liquid TRF (70%) plus surfactants mixture the solid carriers were holding, and the flow of powder remained good to excellent. The impairment in powder flowability properties when a liquid is adsorbed onto carriers with low specific surface area might be due to the high amount of liquid being exposed on the outer surface of the particles, which resulted in interparticulate liquid bonding and increased cohesion [64,65]. Neusilin is an excipient with high specific surface area, and the powder flow was less affected by the addition of liquid. Another reason for the maintenance of good flowability from this formulation might be that the liquid mixture added contained poloxamer that is solid at room temperature, thus reducing the liquid load added to the solid carrier.

The release study was conducted to determine if the adsorbed active ingredient could be released for absorption. From the results obtained, the tocotrienols could be desorbed from Neusilin. This can be attributed to the improved wetting of the solid, which accelerated medium penetration into the voids and capillaries of these solid particles [66]. The high specific surface area and very porous structure of Neusilin might contribute to the homogeneity and fine dispersion of the adsorbed liquid, which enabled higher surface contact with medium or physiological solution [67]. The release of tocotrienols in pH 1.2 appeared to be lower than that in pH 4.5 and 6.8. It has been reported that lipid-based formulations demonstrated impaired release when tested in acidic medium or simulated gastric fluids [68]. This might be due to the fatty acids in the lipid used in the formulation, which are less soluble in highly acidic solution. The TRF used as the oily liquid compound consisted of 45% palm olein that was composed of both saturated and unsaturated fatty acids [69], which could be the reason for the lower release of mixed tocotrienols in pH 1.2 observed in the present study.

There have been reports of an incomplete release of adsorbed compounds from Neusilin [25,63], while some studies reported a complete content release [30,64,67]. Williams et al. [70] reported that excipients in the formulations affect the content release, with higher release observed in more hydrophilic lipid-based formulations. Increasing the surfactant content improved the wetting of Neusilin particles due to the lowering of interfacial tension between formulation and the hydration medium. Surfactants may improve the capillarity of the hydration medium, leading to better penetration of medium into the Neusilin porous structure and increasing the content release [70]. The formulations in the present study used hydrophilic combination surfactants, with addition of excipients that improve wettability and hydration. This could contribute to the good results from the release study observed.

The physical stability of the formed emulsion products that was evaluated based on visual inspection upon standing for 2 h was considered adequate. Emulsion products that were stable (without creaming or phase separation) for 2 h should be able to remain sufficiently dispersed and ready for absorption under the peristaltic movements of the gastrointestinal tract. The emulsion products investigated showed satisfactory physical stability over 2 h. Sediments at the bottom were expected, as Neusilin is insoluble in water.

Emulsion droplet size is critical in determining the rate and extent of drug release and absorption [5,56]. Surfactants have the ability to reduce emulsion droplet size, as the existence of surfactants at the oil and water interface increases the stability of the droplets formed [52]. The addition of surfactants also helped in preventing emulsion droplets from aggregation and maintaining a low polydispersity index value [53]. Formulations that contained only Labrasol^®^ produced emulsions of larger droplet sizes compared to a combination of surfactant or poloxamer-only products. This might be due to its lower HLB value of Labrasol^®^ and the presence of small fraction of mono-, di-, and triglycerides in the product [71].

The optimal droplet size range is recommended to be between 100 and 500 nm [72,73], and the results from all samples investigated fall within this range. It has also been reported that if an emulsion product is in the submicron size, digestion plays a lesser role in drug absorption as compared to a crude emulsion [74]. However, further size reduction in the submicron range (e.g., from 250 to 100 nm) might not be significant in influencing drug absorption [75,76]. Achieving a lower emulsion droplet size is possible with the addition of more surfactants. However, the lowest amount possible of surfactants should be used to prepare an optimal formulation to prevent gastric irritation, which is likely when a high amount of surfactants is used, and to allow more active compounds to be carried per unit mass of the formulation.

Liquid SEDDS are associated with storage instability including drug and/or excipient precipitation or separation and liquid leakage out of capsule shells over time [9,17]. Studies reported that the s-SEDDS has improved stability over time compared to conventional liquid SEDDS [77,78]. From the literature, only a small number of solid tocotrienol products are available in the market and were used in studies, with no specific indication of them being an s-SEDDS preparation [79,80,81]. The stability of tocotrienols is reportedly affected by several environmental factors including rates of lipid oxidation in the matrix, types and concentration of tocopherols, and the presence of other minor compounds such as free fatty acids, water, and minerals [82]. One patent on tocotrienol powder obtained via spray drying showed that 90% of the original tocotrienol content remained after 3 months of storage at 40 °C and relative humidity of 75% [83]. This might be due to embedding of the actives in the solid matrix and limiting their exposure to oxidisation and degradation. Comparing to other tocotrienol powder products, the present study showed that the adsorption method on a solid carrier could provide an alternative approach to obtain a solid tocotrienol powder.

### 4.4. In Vivo Oral Bioavailability Studies

#### 4.4.1. In Vivo Evaluation of Different s-SEDDS Mixed Tocotrienol Formulations

From the plasma concentration profiles obtained with the three s-SEDDS formulations, a spring-parachute effect was observed with B2 for delta- and gamma-tocotrienols, but it was less obvious for the alpha-isoform. This discrepancy could be due to the more lipophilic nature of alpha-tocotrienol, which has been shown to possess better bioavailability over the former two isoforms [41]. However, such a spring-parachute effect was not observed with formulations B1 and B3, suggesting that a mixture of Labrasol^®^ and poloxamer was more effective than using either of the two surfactants alone. This may be due to the better solubilising effect when the two surfactants were combined.

Combining surfactants may improve the emulsification efficiency, as two different surfactants are working together to maximise the solubilisation process of the compound instead of one, thereby increasing its rapid dispersion and allowing absorption to occur [84,85]. The ability of the formulation with combined surfactants to generate emulsion was greatly increased, with higher oil composition compared to when only a single surfactant was used. Higher oil composition would allow higher drug loading to be incorporated [31]. In addition, the combined usage of surfactants may contribute to a better hydrophilic–lipophilic balance (HLB), as the ability of the combined surfactants to partition into the oil–water interface was enhanced [55].

The presence of surfactants has been reported to cause changes in the membrane permeability of the gastrointestinal tract, which could lead to the enhancement of absorption [84,86]. Studies showed that Labrasol^®^ was able to loosen tight junctions and increase permeability, thus enhancing drug absorption by the paracellular pathway [87]. On the other hand, poloxamer was reported to exert inhibitory activity on P-glycoprotein, thus reducing the efflux of absorbed molecules and enhancing the oral bioavailability [86]. Tocotrienols were reported to be substrates of P-glycoprotein [74]. Formulations with a combination of these surfactants may also help to enhance the absorption via these two effects, namely enhancing membrane permeability and inhibiting the P-gp activities. Permeation through the gastrointestinal tract might be easier with the combined effects from both surfactants, resulting in formulation B2 achieving a higher absorption rate compared to the other two formulations that contained either one of the two surfactants only.

All three formulations showed comparable AUC_0-12h_ values, suggesting that the extent of bioavailability of the tocotrienols was not affected by using the surfactants alone or in combination. The difference lies only in the rate of absorption.

#### 4.4.2. In Vivo Evaluation of s-SEDDS Versus Liquid SEDDS and Non-Self-Emulsifying Preparations of Tocotrienols

It is evident that both solid and liquid SEDDS were able to improve the oral bioavailability of tocotrienols. Several factors could contribute to improvement in the oral bioavailability of tocotrienols. Upon the ingestion of the SEDDS, emulsions were spontaneously formed due to the addition of surfactants in the formulations and the agitation provided by the gut motility, effectively presenting the tocotrienols in small emulsion droplets and maintaining the molecules in solubilised form with increased interfacial surface area. Drug exposure during transport to the gastrointestinal membrane was enhanced, therefore improving the absorption and bioavailability [27,32]. The SEDDS were able to ensure that a higher amount of solubilised molecules were readily available for lymphatic transport through intestinal transcellular pathways, which is one of the transportation pathways for lipophilic molecules [88]. As such, the s-SEDDS, B2, exhibited improved tocotrienol bioavailability compared to that of the oily non-self-emulsifying preparation.

The s-SEDDS, B2, appeared to have slightly lower AUC and C_max_ values compared to the liquid SEDDS Tocovid Suprabio™. Several studies had demonstrated a lower oral bioavailability when comparing s-SEDDS with liquid SEDDS [28,29,33,89,90,91]. Liquid SEDDS usually showed a greater initial rate of absorption compared to s-SEDDS due to the liquid SEDDS being administered in a liquid solution state, whereby the release, dispersion, and emulsification were instantaneous [88]. On the other hand, s-SEDDS showed a slower release mechanism compared to the liquid ones. When the solids were exposed to the fluids in the gastrointestinal tract, additional steps including wetting and content release were required to happen before emulsification and dissolution could follow [90]. The delay in the rate of absorption could also be due to the diffusion path length of molecules in the matrix of the porous solid carriers [90].

Van Speybroeck et al. [25] reported that using Neusilin as a solid carrier resulted in a lower bioavailability compared to the corresponding liquid SEDDS. This trend was observed in the present study for delta- and gamma-tocotrienol, but interestingly, not with alpha-tocotrienol. It is apparent that alpha-tocotrienol showed similar levels between the two SEDDS. This suggests that there might be a different affinity of adsorption on Neusilin, which was not demonstrated in the in vitro test.

The commercial preparation Tocovid Suprabio™ used in this study contained 50 mg of mixed tocotrienols in 600 mg capsules, with a composition as follows: 24.8% Tocomin^®^ 50 (a tocotrienol-rich oily suspension), 58.6% palm or soya oil, 14.5% Labrasol^®^, and 2.1% Tween 80 [92]. Direct comparison can be applied only to a certain extent, as its contents and excipients were different compared to the s-SEDDS (powder B2) investigated. Tocovid Suprabio™ has 58.6% of oil, apart from the tocotrienol-rich oily suspension, whereas the s-SEDDS B2 has no addition of extra oil other than those from TRF. The addition of oil might have contributed to the further increment in oral bioavailability due to the effect from bile salts and lipolysis by pancreatic enzymes, which further help to solubilise the lipophilic drug for absorption [74,93].

The non-self-emulsifying oily preparation was prepared by the addition of soya oil to replace the component of surfactants. There appears to be some level of absorption from the non-self-emulsifying oily preparation. This might be due to the unique physiology of rats where bile secretion was continuous [93]. Bile act as endogeneous emulsifier in promoting the solubilisation of the lipophilic molecules [28]. Due to the lipid content in the oily tocotrienol preparation, some emulsification and formation of mixed micelles might have occurred, resulting in the observed oral bioavailability of the tocotrienols.

## 5. Conclusions

In summary, s-SEDDS containing 70% TRF were successfully developed using a simple adsorption method. The formulations showed good self-emulsification efficiency, excellent powder flowability, satisfactory content release from carriers, and small emulsion droplet size. The oral bioavailability of the delta-, gamma-, and alpha-tocotrienol determined from an animal study using adult male Sprague–Dawley rats showed that the s-SEDDS powder with combined surfactants of poloxamer and Labrasol^®^ had a faster rate of absorption compared to that of the formulations prepared with one surfactant (poloxamer or Labrasol^®^ only), despite the extent of bioavailability being similar among the three formulations tested. Further in vivo study carried out using adult male Sprague–Dawley rats showed that the s-SEDDS mixed tocotrienol powder with combined surfactants demonstrated enhanced oral bioavailability compared to non-self-emulsifying liquid TRF when administered at fasted state. The s-SEDDS may serve as a potential alternative to the conventional liquid SEDDS with the added advantage of being a solid dosage form.

## Figures and Tables

**Table 1:** Sequence of administration for Study I and II.

Study	Group	Sequence of Administration		
		Week 1	Week 2	Week 3
I	1	B2	B3	B1
	2	B1	B2	B3
	3	B3	B1	B2
II	1	TRF	B2	Tocovid Suprabio™
	2	Tocovid Suprabio™	TRF	B2
	3	B2	Tocovid Suprabio™	TRF

The s-SEDDS preparations B1, B2, and B3 containing 70% tocotrienol-rich fraction (TRF) and 30% surfactant(s). Formulations B1 contained 30% Labrasol^®^; B2 contained 15% Labrasol^®^ and 15% poloxamer; and B3 contained 30% poloxamer.

**Table 2:** Visual grading results and emulsification time (seconds) in mean (SD) for n = 3 for formulations containing various compositions of tocotrienol-rich fraction (TRF) and surfactant Poloxamer (Pol) and Labrasol^®^ (Lab).

	Composition of Liquid Mixture (%)			Type of Formulation Tested, Visual Grading Results * and Emulsification Time (Seconds)	
Code	TRF	Pol	Lab	(1) Liquid Mixture	(2) Solid Formulation
A1	60	0	40	A, 4.5 (0.4)	A, 5.9 (0.2)
A2	60	20	20	A, 14.4 (0.9)	A, 6.3 (0.7)
A3	60	40	0	C	A, 7.0 (0.9)
B1	70	0	30	C	A, 5.0 (0.6)
B2	70	15	15	A, 11.5 (0.9)	A, 5.2 (0.4)
B3	70	30	0	D	A, 6.8 (0.7)
C1	80	0	20	D	C
C2	80	10	10	D	C
C3	80	20	0	D	A, 13.3 (0.8)

* Visual grading system adapted from Khoo et al. [44].

**Table 3:** Bulked and tapped density, Carr’s compressibility index (CI), Hausner ratio (HR), angle of repose of the s-SEDDS containing 70% TRF and 30% mixed surfactants of Poloxamer and Labrasol^®^. Values in brackets indicate mean (SD) for n = 3.

Formulation	Density (g/mL)		CI (%)		HR		Angle of Repose (°)	
Code	Bulk	Tapped	Value	Class	Value	Class	Value	Class
B1	0.4765	0.5179	7.94	Excellent	1.09	Excellent	31.71	Good
	(0.0002)	(0.0153)	(2.75)		(0.03)		(1.51)	
B2	0.4415	0.4658	5.17	Excellent	1.05	Excellent	27.52	Excellent
	(0.0114)	(0.0192)	(1.43)		(0.02)		(0.94)	
B3	0.4169	0.4415	5.56	Excellent	1.06	Excellent	25.86	Excellent
	(0.0001)	(0.0056)	(1.20)		(0.01)		(0.97)	

**Table 4:** Physical stability, emulsion droplet size (Z-average), and polydispersity index (PDI) of the emulsion formed from the s-SEDDS formulations containing 70% TRF and 30% mixed surfactants of Poloxamer and Labrasol^®^. Values in brackets indicate mean (SD) for n = 3.

Formulation	Physical Stability	Z-Average	PDI
Code		(nm)	
B1	No separation	276.9 (10.7)	0.528 (0.065)
B2	No separation	226.1 (2.4)	0.441 (0.035)
B3	Slight creaming	210.8 (7.2)	0.373 (0.009)

**Table 5:** Pharmacokinetic parameters of delta-, gamma-, and alpha-tocotrienol (n = 6) after oral administration of 20 mg/kg mixed tocotrienols contained in solid formulation B1 (Labrasol^®^ as surfactant), B3 (poloxamer as surfactant), and B2 (Labrasol^®^ and poloxamer as surfactants).

	Delta-Tocotrienol			Gamma-Tocotrienol			Alpha-Tocotrienol		
Formulations	B1	B3	B2	B1	B3	B2	B1	B3	B2
C_max_ (ng/mL)	101.1	81.3	194.9	340.3	249.7	594.2	1013.3	678.1	955.3
	(41.3)	(30.3) *	(84.6)	(104.9)	(60.5) *	(230.8)	(224.3) ^	(89.2) *	(133.9)
T_max_ (h)	9.3	2.3	1.9	9.3	3.3	1.9	9.3	7.3	3.2
	(3.3) *^	(1.9)	(0.6)	(3.3) *	(2.9)	(0.6)	(3.3) *	(1.0) *	(0.8)
AUC_0-12h_ (h.ng/mL)	566.7	500.3	593.7	1895.1	1640.5	1832.0	5745.3	5624.8	5684.5
	(254.0)	(140.6)	(107.6)	(675.1)	(399.5)	(266.2)	(1951.1)	(852.5)	(396.4)
C.I. C_max_	0.36–0.91	0.33–0.63		0.40–0.93	0.35–0.59		0.87–1.36	0.61–0.83	
C.I. AUC_0-12h_	0.66–1.28	0.74–0.99		0.77–1.36	0.80–1.01		0.83–1.18	0.92–1.07	

All the values shown are mean (SD). * *p <* 0.05 when compared to B2. ^ *p <* 0.05 when compared to B3. C.I. C_max_ is the 90% confidence interval of the C_max_ values of B1 and B3 over those of B2. C.I. AUC_0-12h_ is the 90% confidence interval of the AUC_0-12h_ values of B1 and B3 over those of B2.

**Table 6:** Pharmacokinetic parameters of delta-, gamma-, and alpha-tocotrienol (n = 5) after oral administration of 20 mg/kg mixed tocotrienols in B2 (s-SEDDS tocotrienol preparation), Tocovid Suprabio™, and a non-self-emulsifying mixed tocotrienols oily preparation.

	Delta-Tocotrienol			Gamma-Tocotrienol			Alpha-Tocotrienol		
Formulations	B2	Tocovid	TRF	B2	Tocovid	TRF	B2	Tocovid	TRF
C_max_ (ng/mL)	133.5	498.3	57.5	424.3	648.8	192.0	1126.4	1089.7	394.7
	(55.1) ^	(86.3) *	(53.8)	(155.4)	(114.5)	(171.5)	(173.5) ^#^	(123.6) ^#^	(159.3)
T_max_ (h)	4.4	1.8	6.6	4.4	1.8	6.7	6.2	3.0	7.2
	(3.3)	(0.3)	(5.3)	(3.3)	(0.3)	(5.1)	(4.0)	(0.0)	(4.6)
AUC_0-12h_ (h.ng/mL)	665.5	1583.2	228.4	2128.6	2301.3	759.4	7913.4	7509.3	2639.2
	(144.0) *^	(242.0) *	(137.6)	(428.6) *	(252.1) *	(401.6)	(1253.7) *	(571.7) *	(1342.2)
C.I. C_max_	2.25–4.91	8.46–15.62		2.09–4.61	3.24–5.71		2.61–3.32	2.48–3.50	
C.I. AUC_0-12h_	2.57–5.02	6.61–10.50		2.40–4.59	2.78–4.32		2.77–4.09	2.71–3.96	

All the values shown are mean (SD). * *p <* 0.05 when compared to TRF. ^ *p <* 0.05 when compared to Tocovid Suprabio™. # *p <* 0.01 when compared to TRF. C.I. C_max_ is the 90% confidence interval of the C_max_ values of powder B2 and Tocovid Suprabio™ over those of TRF. C.I. AUC_0-12h_ is the 90% confidence interval of the AUC_0-12h_ values of powder B2 and Tocovid Suprabio™ over those of TRF.

## Data Availability

Not applicable.

## Supplementary Materials

The following are available online at https://www.mdpi.com/article/10.3390/pharmaceutics13111777/s1, Figure S1: HPLC chromatogram for delta-, gamma-, and alpha-tocotrienol from in vitro assay. Figure S2: HPLC chromatogram for delta-, gamma-, and alpha-tocotrienol (T3) from in vivo assay. Figure S3: The s-SEDDS preparations B2 containing 70% TRF, 15% Labrasol^®^, and 15% poloxamer. Tables S1–S3: Individual numerical values for C_max_, T_max_, and AUC_0-12h_ (n = 6) for tocotrienols after oral administration of 20 mg/kg mixed tocotrienols in solid formulation B1 (contained Labrasol^®^ as surfactant), B3 (contained poloxamer as surfactant), and B2 (contained Labrasol^®^ and poloxamer as surfactants). Tables S4–S6: Individual numerical values for C_max_, T_max_, and AUC_0-12h_ (n = 5) for tocotrienols after oral administration of 20 mg/kg mixed tocotrienols in B2 (solid self-emulsifying tocotrienol preparation), Tocovid Suprabio™, and a non-self-emulsifying mixed tocotrienols oily preparation.
