# Supplementary material for: Formulation and In Vivo Evaluation of a Solid Self-Emulsifying Drug Delivery System Using Oily Liquid Tocotrienols as Model Active Substance
Source: Pharmaceutics. 2021 Oct 25;13(11):1777. doi: 10.3390/pharmaceutics13111777 (PMC8621674; doi:10.3390/pharmaceutics13111777)
Supplement: Supplementary file 1 [file pharmaceutics-13-01777-s001.zip › pharmaceutics-1371967-supplementary-table s1-need revise.pdf]

pharmaceutics

Supplementary Materials: Formulation and In Vivo Evaluation of a Solid Self-Emulsifying Drug Delivery System Using Oily Liquid Tocotrienols as Model Active Substance

You Zhuan Lee, Eng Kwong Seow, Sheau Chin Lim, Kah Hay Yuen and Nurzalina Abdul Karim Khan

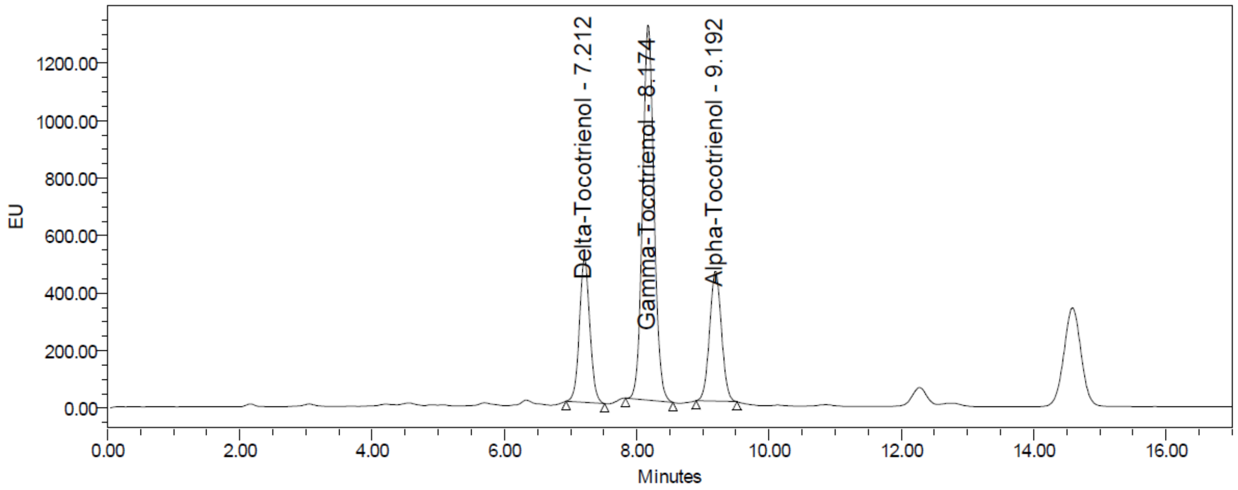

Figure S1. HPLC chromatogram for delta- gamma- and alpha-tocotrienol from in vitro assay.

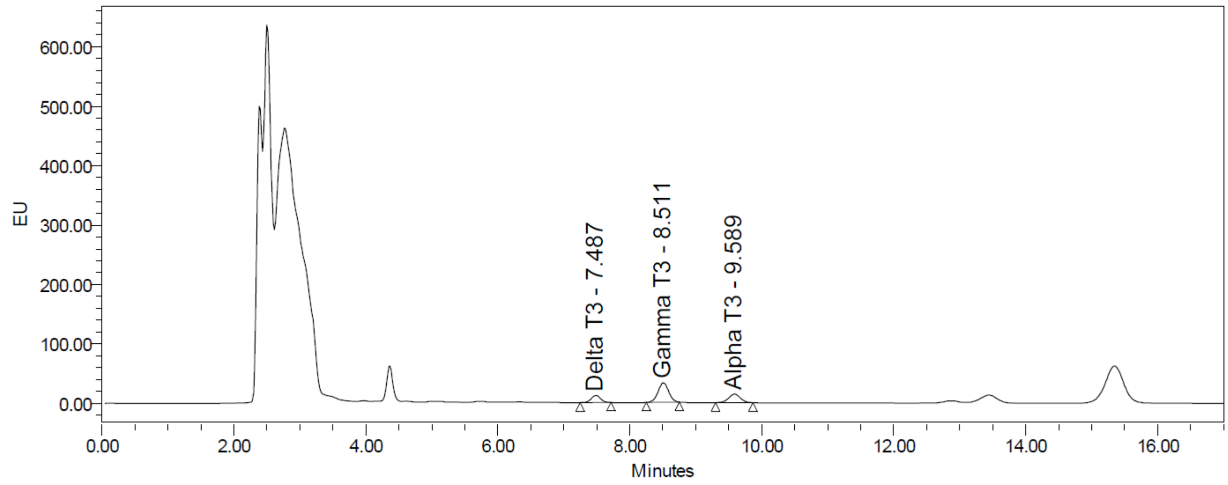

Figure S2. HPLC chromatogram for delta- gamma- and alpha-tocotrienol (T3) from in vivo assay.

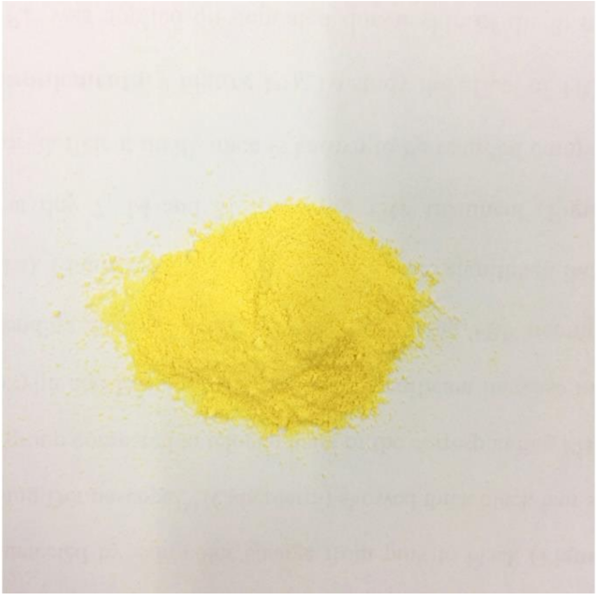

Figure S3. The s-SEDDS preparations B2 containing 70% TRF, 15% Labrasol® and 15% poloxamer.

Table S1 Individual numerical values for C_{max}, T_{max} and AUC_{0-12h} (n = 6) for delta-tocotrienol after oral administration of 20 mg/kg mixed-tocotrienols in solid formulation B1 (contained Labrasol® as surfactant), B3 (contained poloxamer as surfactant) and B2 (contained Labrasol® and poloxamer as surfactants).

	B1			B3			B2		
**Subject**	C_{max} (ng/ml)	T_{max} (h)	AUC_{0-12h} (h.ng/ml)	C_{max} (ng/ml)	T_{max} (h)	AUC_{0-12h} (h.ng/ml)	C_{max} (ng/ml)	T_{max} (h)	AUC_{0-12h} (h.ng/ml)
1	109.0	8.0	811.7	74.0	1.5	537.2	205.4	3.0	708.3
2	48.4	12.0	372.2	86.4	6.0	520.3	355.6	1.5	720.2
3	107.5	12.0	557.1	131.8	1.0	733.4	152.6	2.0	611.7
4	56.1	12.0	175.4	59.9	1.5	409.6	178.8	1.5	524.1
5	151.7	8.0	813.3	91.7	2.0	486.1	165.1	1.5	552.1
6	133.7	4.0	670.7	43.8	1.5	314.9	111.8	2.0	446.1
Mean	101.1	9.3 [a,b]	566.7	81.3 [a]	2.3	500.3	194.9	1.9	593.7
SD	41.3	3.3	254.0	30.3	1.9	140.6	84.6	0.6	107.6
CV%	40.9	35.0	44.8	37.3	82.9	28.1	43.4	30.5	18.1
C.I.	0.36–0.91		0.66–1.28	0.33–0.63		0.74–0.99			
C.I.*	0.92–1.94		0.79–1.60						

[a] $p < 0.05$ when compared to B2

[b] $p < 0.05$ when compared to B3

C.I. is the 90% confidence interval for the ratio of C_{max} and AUC_{0-12h} values of B1 or B3 over those of B2

C.I.* is the 90% confidence interval for the ratio of C_{max} and AUC_{0-12h} values of B1 over those of B3

Table S2 Individual numerical values for C_{max}, T_{max} and AUC_{0-12h} (n = 6) for gamma-tocotrienol after oral administration of 20 mg/kg mixed-tocotrienols in solid formulation B1 (contained Labrasol® as surfactant), B3 (contained poloxamer as surfactant) and B2 (contained Labrasol® and poloxamer as surfactants).

	B1			B3			B2		
**Subject**	C_{max} (ng/ml)	T_{max} (h)	AUC_{0-12h} (h.ng/ml)	C_{max} (ng/ml)	T_{max} (h)	AUC_{0-12h} (h.ng/ml)	C_{max} (ng/ml)	T_{max} (h)	AUC_{0-12h} (h.ng/ml)
1	349.6	8.0	2611.0	237.7	8.0	1689.3	578.7	3.0	1997.7
2	194.9	12.0	1360.5	257.7	6.0	1649.0	1031.9	1.5	2102.4
3	384.7	12.0	1973.7	341.4	1.0	2336.7	478.6	2.0	2021.9
4	232.2	12.0	839.0	231.8	1.5	1544.4	618.2	1.5	1859.6
5	450.3	8.0	2438.5	273.6	2.0	1518.3	482.5	1.5	1546.6
6	429.9	4.0	2148.0	156.0	1.5	1105.1	375.2	2.0	1463.8
Mean	340.3	9.3[a]	1895.1	249.7[a]	3.3	1640.5	594.2	1.9	1832.0
SD	104.9	3.3	675.1	60.5	2.9	399.5	230.8	0.6	266.2
CV%	30.8	35.0	35.6	24.2	87.8	24.4	38.8	30.5	14.5
C.I.	0.40–0.93		0.77–1.36	0.35–0.59		0.80–1.01			
C.I.*	1.06–1.88		0.87–1.56						

[a] $p < 0.05$ when compared to B2

[b] $p < 0.05$ when compared to B3

C.I. is the 90% confidence interval for the ratio of C_{max} and AUC_{0-12h} values of B1 or B3 over those of B2

C.I.* is the 90% confidence interval for the ratio of C_{max} and AUC_{0-12h} values of B1 over those of B3

Table S3 Individual numerical values for C_{max}, T_{max} and $AUC_{0\text{-}12h}$ (n = 6) for alpha-tocotrienol after oral administration of 20 mg/kg mixed-tocotrienols containing in solid formulation B1 (contained Labrasol® as surfactant), B3 (contained poloxamer as surfactant) and B2 (contained Labrasol® and poloxamer as surfactants).

Subject	B1			B3			B2		
	C_{max} (ng/ml)	T_{max} (h)	$AUC_{0\text{-}12h}$ (h.ng/ml)	C_{max} (ng/ml)	T_{max} (h)	$AUC_{0\text{-}12h}$ (h.ng/ml)	C_{max} (ng/ml)	T_{max} (h)	$AUC_{0\text{-}12h}$ (h.ng/ml)
1	1180.9	8.0	9172.2	816.7	8.0	6000.2	989.9	3.0	5950.9
2	854.8	12.0	5022.7	594.4	6.0	4701.6	1149.4	2.0	5265.8
3	1391.3	12.0	5112.3	687.3	6.0	5997.3	805.3	4.0	6248.4
4	863.9	12.0	3252.5	693.2	8.0	6809.9	1060.4	3.0	5710.9
5	943.9	8.0	6136.6	709.6	8.0	5661.9	849.8	4.0	5715.7
6	844.8	4.0	5775.4	567.2	8.0	4578.0	877.3	3.0	5215.6
Mean	1013.3 [b]	9.3 [a]	5745.3	678.1 [a]	7.3 [a]	5624.8	955.3	3.2	5684.5
SD	224.3	3.3	1951.1	89.2	1.0	852.5	133.9	0.8	396.4
CV%	22.1	35.0	34.0	13.2	14.1	15.2	14.0	23.8	7.0
C.I.	0.87–1.36		0.83–1.18	0.61–0.83		0.92–1.07			
C.I.*	1.36–1.66		0.85–1.22						

[a] $p < 0.05$ when compared to B2

[b] $p < 0.05$ when compared to B3

C.I. is the 90% confidence interval for the ratio of C_{max} and $AUC_{0\text{-}12h}$ values of B1 or B3 over those of B2

C.I.* is the 90% confidence interval for the ratio of C_{max} and $AUC_{0\text{-}12h}$ values of B1 over those of B3

Table S4 Individual numerical values for C_{max}, T_{max} and AUC_{0-12h} (n = 5) for delta-tocotrienol after oral administration of 20 mg/kg mixed-tocotrienols in B2 (solid self-emulsifying tocotrienol preparation), Tocovid Suprabio™, and a non self-emulsifying mixed-tocotrienols oily preparation (TRF).

	B2			Tocovid Suprabio™			TRF		
**Subject**	C_{max} (ng/ml)	T_{max} (h)	AUC_{0-12h} (h.ng/ml)	C_{max} (ng/ml)	T_{max} (h)	AUC_{0-12h} (h.ng/ml)	C_{max} (ng/ml)	T_{max} (h)	AUC_{0-12h} (h.ng/ml)
1	103.8	8.0	619.2	360.3	2.0	1175.7	21.2	12.0	135.8
2	89.2	8.0	685.2	530.0	1.5	1710.7	152.7	1.0	435.0
3	188.0	2.0	870.5	477.2	2.0	1625.5	34.0	12.0	120.7
4	198.6	2.0	682.2	586.3	1.5	1806.5	43.4	6.0	305.5
5	87.8	2.0	470.3	537.8	2.0	1597.5	36.2	2.0	145.2
Mean	133.5 [b]	4.4	665.5 [a,b]	498.3 [a]	1.8	1583.2 [a]	57.5	6.6	228.4
SD	55.1	3.3	144.0	86.3	0.3	242.0	53.8	5.3	137.6
CV%	41.3	74.7	21.6	17.3	15.2	15.3	93.6	79.9	60.2
C.I.	2.25−4.91		2.57−5.02	8.46−15.62		6.61−10.50			
C.I. *	0.20−0.34		0.36−0.50						

[a] $p < 0.05$ when compared to TRF

[b] $p < 0.05$ when compared to Tocovid Suprabio™

C.I. is the 90% confidence interval for the ratio of C_{max} and AUC_{0-12h} values of solid powder B2 and Tocovid Suprabio™ over those of TRF

C.I.* is the 90% confidence interval for the ratio of C_{max} and AUC_{0-12h} values of solid powder B2 over those of Tocovid Suprabio™

Table S5 Individual numerical values for C_{max}, T_{max} and AUC_{0-12h} (n = 5) for gamma-tocotrienol after oral administration of 20 mg/kg mixed-tocotrienols in B2 (solid self-emulsifying tocotrienol preparation), Tocovid Suprabio™, and a non self-emulsifying mixed-tocotrienols oily preparation (TRF).

	B2			Tocovid Suprabio™			TRF		
**Subject**	C_{max} (ng/ml)	T_{max} (h)	AUC_{0-12h} (h.ng/ml)	C_{max} (ng/ml)	T_{max} (h)	AUC_{0-12h} (h.ng/ml)	C_{max} (ng/ml)	T_{max} (h)	AUC_{0-12h} (h.ng/ml)
1	370.0	8.0	2264.8	468.2	2.0	1865.3	83.1	12.0	498.7
2	275.7	8.0	2239.4	647.2	1.5	2511.9	496.3	1.5	1376.9
3	579.8	2.0	2709.7	634.1	2.0	2335.5	107.6	12.0	441.6
4	600.1	2.0	1822.2	758.4	1.5	2384.9	138.0	6.0	956.9
5	296.1	2.0	1606.7	736.0	2.0	2408.9	135.3	2.0	523.0
Mean	424.3	4.4	2128.6 [a]	648.8	1.8	2301.3 [a]	192.0	6.7	759.4
SD	155.4	3.3	428.6	114.5	0.3	252.1	171.5	5.1	401.6
CV%	36.6	74.7	20.1	17.7	15.2	11.0	89.3	76.8	52.9
C.I.	2.09−4.61		2.40−4.59	3.24−5.71		2.78−4.32			
C.I. *	0.49−0.82		0.79−1.10						

[a] $p < 0.05$ when compared to TRF

C.I. is the 90% confidence interval for the ratio of C_{max} and AUC_{0-12h} values of solid powder B2 and Tocovid Suprabio™ over those of TRF

C.I.* is the 90% confidence interval for the ratio of C_{max} and AUC_{0-12h} values of solid powder B2 over those of Tocovid Suprabio™

Table S6 Individual numerical values for C_{max}, T_{max} and AUC_{0-12h} (n = 5) for alpha-tocotrienol after oral administration of 20 mg/kg mixed-tocotrienols in B2 (solid self-emulsifying tocotrienol preparation), Tocovid Suprabio™, and a non self-emulsifying mixed-tocotrienols oily preparation (TRF).

	B2			Tocovid Suprabio™			TRF		
Subject	C_{max} (ng/ml)	T_{max} (h)	AUC_{0-12h} (h.ng/ml)	C_{max} (ng/ml)	T_{max} (h)	AUC_{0-12h} (h.ng/ml)	C_{max} (ng/ml)	T_{max} (h)	AUC_{0-12h} (h.ng/ml)
1	1127.9	12.0	8025.2	944.3	3.0	7154.2	369.2	12.0	2303.2
2	1412.9	8.0	9835.4	1139.9	3.0	7445.5	673.6	2.0	4876.0
3	1023.5	6.0	8072.1	973.3	3.0	6929.8	303.1	12.0	1518.6
4	1106.8	2.0	7093.1	1223.1	3.0	8422.7	341.7	6.0	2763.7
5	960.9	3.0	6541.4	1167.8	3.0	7594.5	285.8	4.0	1734.4
Mean	1126.4 [b]	6.2	7913.4 [a]	1089.7 [b]	3.0	7509.3 [a]	394.7	7.2	2639.2
SD	173.5	4.0	1253.7	123.6	0.0	571.7	159.3	4.6	1342.2
CV%	15.4	64.9	15.8	11.3	0.0	7.6	40.4	63.9	50.9
C.I.	2.61−3.32		2.77−4.09	2.48−3.50		2.71−3.96			
C.I. *	0.89−1.18		0.91−1.21						

[a] $p < 0.05$ when compared to TRF

[b] $p < 0.01$ when compared to TRF

C.I. is the 90% confidence interval for the ratio of C_{max} and AUC_{0-12h} values of solid powder B2 and Tocovid Suprabio™ over those of TRF

C.I.* is the 90% confidence interval for the ratio of C_{max} and AUC_{0-12h} values of solid powder B2 over those of Tocovid Suprabio™
